# Cordycepin induces autophagy-mediated c-FLIPL degradation and leads to apoptosis in human non-small cell lung cancer cells

**DOI:** 10.18632/oncotarget.14262

**Published:** 2016-12-27

**Authors:** Xinghui Yu, Jianya Ling, Xianfang Liu, Sen Guo, Yidan Lin, Xiangguo Liu, Ling Su

**Affiliations:** ^1^ Shandong Provincial Key Laboratory of Animal Cells and Developmental Biology, Shandong University School of Life Sciences, Jinan, China; ^2^ The Department of Otolaryngology Head and Neck Surgery, Shandong Provincial Hospital Affiliated to Shandong University, Jinan, China; ^3^ The Thoracic Surgery Department of West China Hospital, West China Medical School of Sichuan University, Chengdu, China

**Keywords:** apoptosis, c-FLIP, autophagy, cordycepin

## Abstract

Cordycepin, a main active composition extracted from *Cordyceps militaris*, has been reported to exert anti-tumor activity in a broad spectrum of cancer types. However, the function of cordycepin on human non-small cell lung cancer cells is still obscure. Our present work showed that cordycepin inhibited cell growth by inducing apoptosis and autophagy in human NSCLC cells. Further study revealed that cordycepin triggered extrinsic apoptosis associated with down-regulation of c-FLIP_L_ which suppresses the activity of caspase-8. And ectopic expression of c-FLIP_L_ dramatically prevented cordycepin-caused apoptosis. Meanwhile, cordycepin stimulated autophagy through suppressing mTOR signaling pathway in lung cancer cells. When autophagy was blocked by Atg5 siRNA or PI3K inhibitor LY294002, the levels of apoptosis caused by cordycepin were obviously attenuated. In addition, suppression of autophagy could also elevate the level of c-FLIP_L_ which indicated cordycepin-triggered autophagy promoted the degradation of c-FLIP_L_. Therefore, we conclude that cordycepin induces apoptosis through autophagy-mediated downregulation of c-FLIP_L_ in human NSCLC cells. Taken together, our findings provide a novel prospect on the anti-tumor property of cordycepin, which may further prompt cordycepin to serve as a promising therapeutic approach in NSCLC treatment.

## INTRODUCTION

Lung cancer has been a disastrous malignant neoplasm with highest incidence and mortality all over the world, which represents a poor five-year survival rate of less than 15% [1]. Hence, it is extraordinarily urgent to develop and exploit novel anticancer agents to improve its clinical outcomes. Nowadays, natural agents have attracted much attention for cancer treatment. Cordycepin (3'-deoxyadenosine), a natural product derived from *Cordyceps sinensis*, has been widely used in Chinese traditional medicine. Cordycepin possesses multiple pharmacological properties, such as anti-fungal, anti-bacterial, anti-inflammatory and anti-tumor effects [2, 3]. And the anti-cancer ability has been observed in various cancer types including leukemia, gallbladder, colon, prostate, breast, hepatic, oral carcinoma and so on [4]. Cordycepin inhibits cancer cell growth through cell cycle arrest and apoptosis induction [5]. Importantly, Ames and subacute toxicity test showed that cordycepin exhibited non-mutagenic and non-toxic property in rat model by oral administration [6]. However, the effects of cordycepin on human NSCLC cells have not been deeply investigated.

There are two main signaling pathways involved in apoptosis: the extrinsic pathway and the intrinsic pathway. The extrinsic pathway also is named death receptor pathway which is mediated by the stimulation of cell surface receptors when bound to particular ligands. Once death receptors are trimerized, death-inducing signaling complex (DISC) is rapidly assembled and leads to the activation of pro-caspase8, thereafter stimulates effector caspases, resulting in apoptosis eventually [7]. Cellular-FLICE inhibitory protein (c-FLIP) is a key anti-apoptotic regulator preventing apoptosis via inhibiting caspase8 activation [8]. The intrinsic pathway, referred to as mitochondrial-mediated pathway, is characterized as increased mitochondrial permeability and release of cytochrome c [9]. Meanwhile, this pathway is tightly regulated by a group of proteins belonging to Bcl-2 family, which consists of pro- and anti-apoptotic proteins. Of note, Bcl-2 and Mcl-1 are well investigated anti-apoptotic members that negatively modulated apoptosis [10].

Autophagy is an evolutionary conserved cellular degradation process by which cytoplasmic components and organelles are packaged into autophagosome, and transported into lysosome for digesting cytoplastic garbage and maintaining cellular homeostasis [11]. This physiological procedure can be activated by diverse cellular stresses, such as nutrient limitation, energy deficiency, oxidative stress, and protein or organelle accumulation [12]. Autophagy possesses dual functions in apoptosis, working as either a suppressor or as a promoter for apoptosis induction [13].

Mammalian target of rapamycin (mTOR) and its modulators are pivotal for cells to sense growth factor, cellular energy and nutrient status, thereby regulates cell growth, protein synthesis and autophagy [12]. The serine/threonine kinase mTOR is composed of two distinctly different compounds: mTORC1 and mTORC2. mTORC1 is the real sensor to rapamycin and controls protein synthesis by two main substrates: p70 ribosomal protein S6-kinase (p70S6K) and eukaryotic initiation factor 4E binding protein 1 (EIF4EBP1) [14]. Previous studies demonstrated that inactivation of mTOR contributed to the induction of autophagy by relieving the blockage of Ulk-1 complex [15].

In this study, we found that cordycepin triggered autophagic flux by suppressing mTOR signaling pathway. Additionally, cordycepin-caused autophagy promoted extrinsic apoptosis by down-regulating c-FLIP_L_ in NSCLC cells. Together, our findings may lay the foundation for cordycepin to develop a novel anticancer agent for tumor treatment.

## RESULTS

### Cordycepin induces caspase-dependent apoptosis in human NSCLC cells

**Figure 1:**
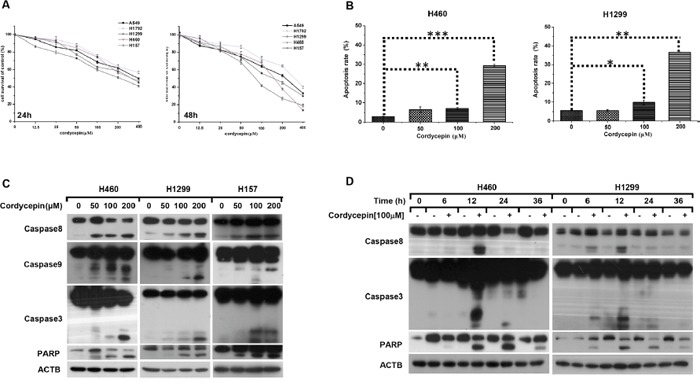
Cordycepin triggers caspase-dependent apoptosis in NSCLC **A**. Cell viability of H1792, A549, H1299, H157 and H460 cells was measured by SRB assay after treated with the indicated concentrations of cordycepin for 24 h and 48 h. **B**. Flow cytometry analysis was performed in H460 and H1299 cell lines after treated with the indicated concentrations of cordycepin for 24 h. The indicated cells were treated with different concentrations of cordycepin for 12 h **C**. or exposed to 100 μM cordycepin for the indicated time **D**., thereafter whole cell lysates were collected for Western blot analysis. Data were normalized to control and represented mean ± SEM for three independent experiments (*p < 0.05, **p < 0.01, ***p < 0.001).

To investigate the effects of cordycepin on cell growth in human lung cancer cells, five NSCLC cell lines (H1792, H1299, H460, H157 and A549) were exposed to increasing concentrations of cordycepin (0, 12.5, 25, 50, 100, 200, 400 μM) for 24 h and 48 h respectively. Then cell viability was measured by SRB assay. As shown in Figure 1A, the survival of NSCLC cells was significantly reduced in a dose-dependent manner after treatment with cordycepin for 24 h. Then we calculated the half-maximal inhibitory concentration (IC50) based on the data from SRB assay and found that the IC50 value of H460, H1299 and H157 cell lines was approximately 200 μM, while H1792 and A549 had a higher IC50 value of 400 μM. Moreover, in comparison with control samples, the viability of H460, H1299 and H157 cell lines were suppressed up to 80% after cordycepin treatment for 48 h, therefore these cell lines were chosen to conduct subsequent analysis.

To illuminate the mechanism that cordycepin caused survival suppression in human NSCLC cells, flow cytometry analysis was performed to examine the impact of cordycepin on apoptosis and the data revealed that cordycepin dramatically increased the percentage of early and late apoptotic cells in H1299 and H460 after treated with increasing dosage of cordycepin for 24 h (Figure 1B). Afterwards, we carried out western blot assay to detect whether cordycepin triggered caspase-dependent apoptosis and found that the cleaved forms of caspase-8, caspase-9, caspase-3 and PARP were significantly elevated both in a dose-dependent manner (Figure 1C). Moreover, the time course assay revealed that the cleavage of caspases was appeared at 6 h, and reached the peak at 12 h (Figure 1D). Therefore, we draw the conclusion that cordycepin leads to caspase-dependent apoptosis in NSCLC.

### c-FLIP_L_ is involved in cordycepin-induced apoptosis

**Figure 2:**
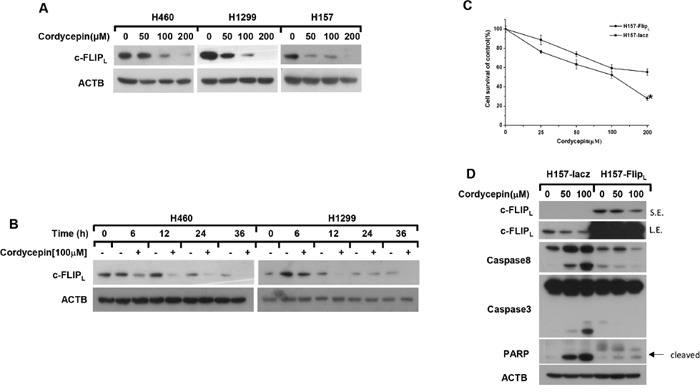
Cordycepin induces apoptosis through down-regulation of c-FLIPL The indicated cell lines were disposed with 0, 50, 100 and 200 μM cordycepin for 12 h **A**. or treated with 100 μM cordycepin for the indicated time **B**., then the whole-cell protein lysates were harvested and prepared for western blot analysis. **C**. The viability of H157-FLIP_L_ and H157-lacz cells were measured by SRB assay after exposed to the indicated concentrations of cordycepin for 24 h. **D**. H157-FLIP_L_ and H157-lacz cells were treated with 0, 50, 100 μM condycepin for 12 h, then whole cell lysates were collected for western blot analysis. Data represented mean ± SEM for three independent experiments each. L.E.: long exposure; S.E.: short exposure. *p < 0.05, **p < 0.01, ***p < 0.001

c-FLIP has been shown to be a key negative regulator of extrinsic apoptosis by inhibiting the activation of caspase-8 in human cancer cells [16]. Thus, we detected whether cordycepin influenced the levels of c-FLIP in NSCLC cells. H1299, H460 and H157 cells were exposed to different concentrations of cordycepin for 12 h or to 100 μM cordycepin for various times, and the results indicated that c-FLIP_L_ was greatly down-regulated in a dose and a time-dependent manner (Figure 2A and 2B). Furthermore, we examined whether c-FLIP_L_ played a critical role in cordycepin-induced extrinsic apoptosis. H157-FLIP_L_ cells stably over-expressed c-FLIP_L_ were used to validate the function of c-FLIP_L_ in cordycepin-induced apoptosis. As shown in Figure 2C, the cell viability of H157-FLIP_L_ was higher than that in H157-lacz cells especially in the concentration of 200 μM. What's more, compared with H157-lacz cells, the cleavage of caspase-8, caspase-3 and PARP was significantly weakened in H157-FLIP_L_ cells (Figure 2D). Taken together, downregulation of c-FLIP_L_ contributes to cordycepin-induced apoptosis in human lung cancer cells.

### Cordycepin triggers autophagy by inhibiting mTOR pathway

**Figure 3:**
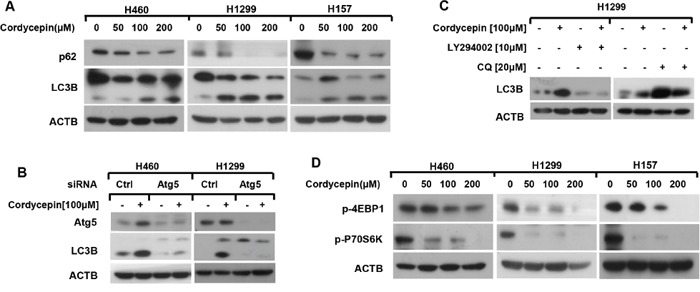
Cordycepin inactivates mTOR and leads to autophagy in NSCLC cells **A** and **D**. H460, H1299 and H157 cell lines were treated with 0, 50, 100 and 200 μM cordycepin for 12 h. **B**. H460 and H1299 cells were conducted with Atg5 siRNA and treated with 100 μM cordycepin for 12 h. **C**. H1299 cells were pretreated with10 μM LY294002 or 20 μM CQ for 1h and then exposed to 100 μM cordycepin for another 12 h. Then whole-cell protein lysates were harvested and prepared for western blot analysis.

Because autophagy plays vital role in determining cell fate [12], we wondered whether cordycepin induced autophagy in NSCLC cells. The conversion of LC3B-I to LC3B-II by conjugating with phosphatidylethanolamine is the typical recognition of autophagy. Thus, we detected the expression of LC3B-II formation after treatment with the indicated concentration of cordycepin for 12 h and found that the conversion of LC3B-II was obviously enhanced in a dose-dependent manner (Figure 3A). Moreover, p62, an autophagy receptor which can bind to specific cargo for degradation in the lysosome during the process of autophagy, was down-regulated in a dose-dependent manner after cordycepin treatment, providing evidence for autophagy occurrence (Figure 3A).

To further supervise the autophagic flux induced by cordycepin, H1299 and H460 cell lines were transfected with Atg5 siRNA to inhibit Atg5 expression and the transformation of LC3B-II was dramatically decreased after Atg5 silence (Figure 3B). Furthermore, when H1299 cells were co-incubated with cordycepin and LY294002, which obstructs the early stage of autophagy, the level of LC3B-II was greatly down-regulated (Figure 3C). On the contrary, co-treatment with cordycepin and chloroquine (CQ), which blocks the late stage of autophagy, enhanced the transformation of LC3B-II (Figure 3C). In conclusion, we deduce that cordycepin induces autophagic flux in human NSCLC cells.

mTOR signaling works as a crucial modulator of autophagy initiation. p-P70S6K and p-4EBP1 exist as the two vital substrates of mTOR [17]. Furthermore, we inspected whether cordycepin-triggered autophagy through mTOR suppression. Human NSCLC cell lines H460, H1299 and H157 were treated with the indicated dosage of cordycepin for 12 h, and the dose-dependent western blot results showed that cordycepin explosion notably declined the levels of p-P70S6K and p-4EBP1 (Figure 3D). Therefore, we speculated that cordycepin induced autophagy via mTOR inhibition.

### Cordycepin causes pro-apoptotic autophagy in NSCLC cells

**Figure 4:**
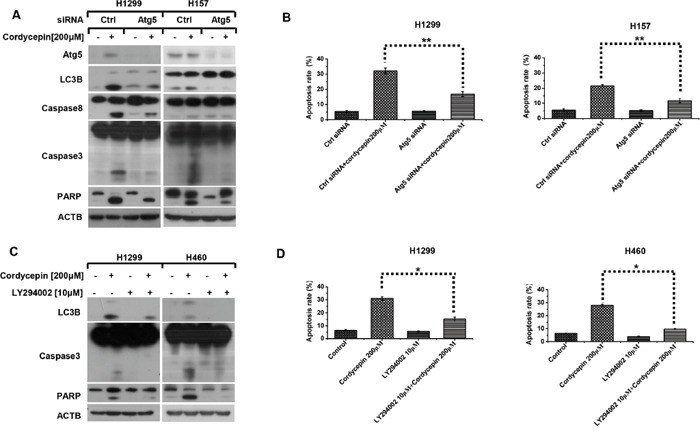
Cordycepin gives rise to pro-apoptotic autophagy in NSCLC cells H1299 and H157 cell lines were seeded in six-well plates and conducted with Atg5 siRNA transfection on the next day. After Forty-eight hours transfection, cells were exposed to 200 μM cordycepin for 12 h. Subsequently, cells were harvested and prepared for western blot analysis **A**. or subjected to flow cytometry analysis after 24 h treatment **B**. H1299 and H460 cell lines were seeded in six-well plates and pretreated with 10 μM LY294002 for 1h on the next day, then cells were exposed to 200 μM cordycepin for 12 h. Subsequently, cells were harvested and prepared for western blot analysis **C**. or subjected to flow cytometry analysis after 24 h treatment **D**. Data represented mean ± SEM from three independent experiments (*p < 0.05, **p < 0.01, ***p < 0.001)

Since cordycepin induces apoptosis and autophagy in human NSCLC cells, we further explore the relationship between cordycepin-triggered apoptosis and autophagy. Atg5 siRNA was employed to inhibit Atg5 expression in NSCLC cells, and then western blot analysis was used to detect apoptosis-related proteins. The data demonstrated that Atg5 silence significantly abrogated cordycepin-caused cleavage of caspase-8, caspase-3 and PARP (Figure 4A). Furthermore, flow cytometry analysis was applied to investigate the effect of autophagy on cordycepin-induced apoptosis. Consistently, we found that inhibiting autophagy effectively blocked cordycepin-induced apoptosis in human lung cancer cells (Figure 4B).

To further confirm the interaction between apoptosis and autophagy by cordycepin, H460 and H1299 were pretreated with LY294002, an early inhibitor of autophagy for 1 h, and then exposed to 200 μM cordycepin for 12 h. According to Figure 4C, co-treatment with LY294002 and cordycepin apparently decreased the cleavage of caspase3 and PARP, which was in accordance with the results of flow cytometry analysis (Figure 4D). Consequently, we come to the conclusion that cordycepin-induced autophagy facilitates apoptosis.

### Cordycepin-triggered autophagy promotes c-FLIP degradation

**Figure 5:**
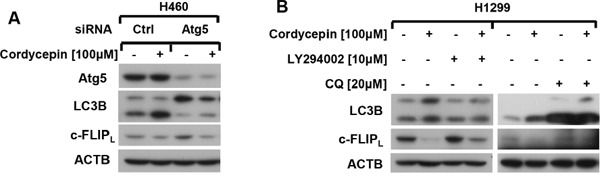
Cordycepin promotes c-FLIP degradation through activating autophagy **A**. H460 cells were seeded in six-well plates and treated with Atg5 siRNA on the next day. After Forty-eight hours transfection, cells were exposed to 100 μM cordycepin for 12 h. Thereafter, cells were harvested and prepared for western blot analysis. **B**. H1299 cells were pretreated with 10 μM LY294002 or 20 μM CQ for 1h, and then exposed to 100 μM cordycepin for another 12 h. The whole-cell protein lysates were harvested and subjected to western blot analysis.

**Figure 6:**
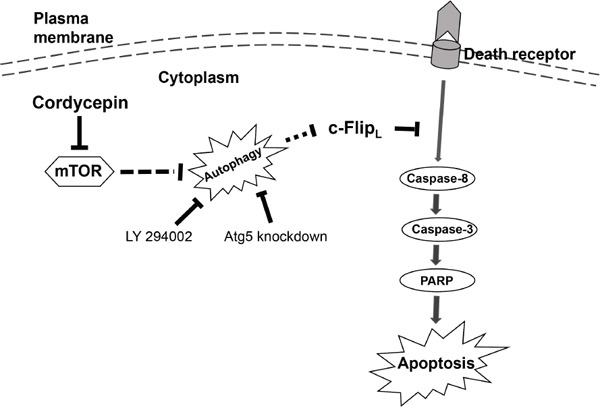
The schematic model of cordycepin-triggered apoptosis and autophagy in human NSCLC cells Cordycepin treatment stimulates extrinsic apoptosis pathway by inhibiting the expression of c-FLIP_L_. Furthermore, cordycepin also triggered autophagy through suppression of mTOR signaling pathway. Cordycepin-induced autophagy promoted apoptosis through degrading c-FLIP_L_.

As cordycepin induced apoptosis through c-FLIP_L_ down-regulation and stimulated pro-apoptotic autophagy in human NSCLC cells, we speculated that cordycepin influenced c-FLIP_L_ degradation by autophagy, which eventually induced apoptosis. As shown in Figure 5A, Atg5 siRNA was utilized to suppress autophagy, and western blot assay revealed that Atg5 knockdown obviously weakened the decrease of c-FLIP_L_ after cordycepin exposure. Moreover, autophagy inhibitors such as LY294002 or CQ, blocked the upstream or downstream steps of autophagy respectively, were used to examine the impact of autophagy on c-FLIP_L_ degradation. The results showed that either blocking the upstream of autophagy by LY294002 or interdicting the downstream of autophagy by CQ, the decrease of c-FLIP_L_ triggered by cordycepin was significantly attenuated (Figure 5B). Hence, we propose that cordycepin treatment triggers autophagy-mediated degradation of c-FLIP_L_, leading to apoptosis eventually in human lung cancer cells (Figure 6).

## DISCUSSION

Recently, many efforts have been done to investigate cordycepin-induced cell death. However, the elaborate molecular mechanism of apoptosis triggered by cordycepin is still poorly deciphered in NSCLC. Thus, the main purposes of our research focused on illuminating the underlying mechanisms of cordycepin-induced apoptosis as well as exploring the relationship between apoptosis and autophagy caused by cordycepin.

It has been reported that cordycepin induces apoptosis through enhancing the expression of JNK, p38 kinase and Bcl-2 pro-apoptotic members [18], increasing the mitochondrial translocation of Bax and the release of cytochrome C, and causing the reactive oxygen species (ROS)-mediated activation of caspases [19]. In our present study, we found that cell viability of human NSCLC cells greatly dropped in both a dose- and a time-dependent fashion after cordycepin incubation, indicating that cordycepin exerted killing effects on human lung cancer cells. In addition, flow cytometery analysis revealed that approximately 40 percentages of H1299 and H460 cells step into early and late phase apoptosis after treated with 200 μM cordycepin for 24 h. And the cleaved forms of caspase-8 (the initiator caspase of extrinsic apoptosis), caspase-9 (the initiator caspase of intrinsic apoptosis), caspase-3 and PARP were also raised in both a dose- and a time-dependent manner. The data suggest that cordycepin induced extrinsic and intrinsic apoptosis in human lung cancer cells.

Furthermore, we inspected the mechanism of cordycepin-induced apoptosis and discovered that the expression of c-FLIP_L_ was dramatically down-regulated, and the ectopic expression of c-FLIP_L_ decreased the cleavage of caspase-8, caspase-3 and PARP, protecting H157-FLIP_L_ cells from cordycepin-triggered extrinsic apoptosis. Our results provide detailed clues to explain cordycepin-induced apoptosis.

Autophagy is a conserved degradation physiological process served to protect cells from the potential damages induced by dysfunctional organelles and protein aggregations [20]. Here, we showed that cordycepin strengthened the transformation of LC3B-II and heightened the degradation of p62, which implied that cordycepin may induce autophagy in lung cancer. Furthermore, to monitor the autophagic flux stimulated by cordycepin, H1299 cells were co-treated with cordycepin and the autophagy inhibitors such as LY294002 and CQ, which blocked the early and the late stages of autophagy respectively. Western blot analysis revealed that the conversion of LC3B-II was greatly decreased after LY294002 co-treatment, while an obvious increase of LC3B-II transformation was occurred after CQ co-incubation. Collectively, these results suggest that cordycepin induces autophagic flux in human lung cancer cells.

mTOR is one of the classical pathways that regulates autophagic induction by two main downstream elements: p70S6K and 4EBP1 [21]. Consistently, western blot assay revealed that the level of p-P70S6K and p-4EBP1 were down-regulated following cordycepin therapy, indicating that mTOR might participate in cordycepin-induced autophagy. mTOR not only negatively regulate autophagy, but also play an important role in regulating cell survival and apoptosis. Thus, we conjectured that cordycepin-caused mTOR suppressing might account for the induction of early autophagy and subsequent apoptosis in NSCLC cells.

As autophagy and apoptosis are two important cellular processes with complex and intersecting protein networks, extensive investigations have focused on the relationship of them [13]. In some circumstance, autophagy develops a stress adaptation by suppressing apoptosis to avoid cell death [22]. Meanwhile it has been reported that inhibition of autophagy promotes apoptosis and decreases cell viability in LNCaP cells after treated with cordycepin [23]. Recently, Munoz-Pinedo et al. proposed a new term-“Autosis”, which is used to define the autophagic cell-death pathway [24]. In our research, we found that inhibiting autophagy by silencing Atg5 expression or LY294002 treatment weakened cordycepin-induced apoptosis, indicating cordycepin triggered pro-apoptotic autophagy in NSCLC cells. However, the molecular mechanism of autophagy exerted pro-apoptotic function in cordycepin-induced cell death is still unclear.

It has been reported that drug-induced autophagy down-regulates c-FLIP and enhances apoptosis in NSCLC cells [25, 26]. Here, we found that cordycepin induced c-FLIP_L_-mediated apoptosis and triggered pro-apoptotic autophagy in human lung cancer cells. Thus, we further investigated the impact of cordycepin-caused autophagy on the degradation of c-FLIP_L_. Western blot analysis showed that the degradation of c-FLIP_L_ was dramatically attenuated when autophagy was blocked by Atg5 knockdown. Moreover, the decrease of c-FLIP_L_ caused by cordycepin was also greatly weakened after autophagy inhibitors (LY294002 and CQ) treatment. Hence, we propose that cordycepin-caused autophagy promotes c-FLIP_L_ down-regulation.

In summary, our results demonstrate that cordycepin triggers autophagy-mediated c-FLIP_L_ degradation and promotes apoptosis in human NSCLC cells. Thus, we illuminate a novel molecular mechanism of apoptosis caused by cordycepin, which may provide theoretical basis for exploitation and application of cordycepin derivatives in cancer treatment.

## MATERIALS AND METHODS

### Reagents and antibodies

Cordycepin (>98% pure) was purchased from Mellon (Dalian China). The stock solution of cordycepin (0.5 mol/L) was prepared in DMSO and diluted to the desired concentration with fresh medium immediately before use. PARP, Caspase-8, Caspase-9, pp70S6K, p-4EBP1 and Atg5 antibodies were purchased from Cell Signaling Technology (Danvers, MA). Mcl-1 and Bcl-2 antibodies were purchased from Santa cruz (Santa Cruz, CA). Caspase-3 antibody was purchased from Imgenex (San Diego, CA). β-actin and LC3B antibodies were purchased from Sigma (Santa Clara, CA). p62 antibody was purchased from Becton, Dickinson and Company (New Jersey, USA)

### Cell lines and cell culture

Human NSCLC cells lines H1792, H1299, H460, H157 and A549 were initially obtained from the American Type Culture Collection (Manassas, VA). Monolayer cultures of all the cell lines were cultivated in RPMI 1640 supplemented with 5% FBS in an atmosphere of 95% air and 5% CO_2_ at 37 °C.

### Cell survival assay

Sulforhodamine B (SRB) assay was used to assess cell survival as described earlier [27]. IC50 (half maximal inhibitory concentration) value was determined by the software SPSS.

### Western blot analysis

The preparation of whole-cell protein lysates and the Western blot analysis were carried out as previously described [28].

### Establishment of stable cell lines that overexpress c-FLIP_L_

c-FLIP_L_ coding regions were amplified by PCR and cloned into the pLenti6 vector (Invitrogen) according to the manufacturer's protocol. 293FT cells were used to prepare lentivirus and the selection of H157 cell clones was according to the procedure previously described [29]. For simplicity, the clone that over-expressed c-FLIP_L_ is referred to as H157-FLIP_L_ and the control clone is named as H157-LacZ.

### Small interference RNA transfection

H1299, H157 and H460 cells were seeded in 6-well plates and treated at the confluency of 50% with a control nonspecific siRNA and Atg5 siRNA using Polyplus transfection reagent according to the manufacturer's recommendations. After 24 h incubation, cells were treated with 200 μM cordycepin and the gene silencing effects were evaluated by western blot analysis. Atg5 siRNA duplexes target the sequences 5'-CCTTTGGCCTAAGAAGAAA-3'.

### Flow cytometric analysis

Cell apoptosis was examined by Annexin V-FITC Apoptosis Detection Kit purchased from Biobox Biotech (Nanjing, China) according to the manufacture's protocol.

### Statistical analysis

Each value is the mean of at least three separate experiments. Student's *t*-test was performed to compare the differences between treated groups and their controls. Difference was considered significant at P<0.05.

## Abbreviations

c-FLIP: cellular FLICE (FADD-like IL-1β-converting enzyme)-inhibitory protein
CQ: Chloroquine
DISC: death-inducing signaling complex
mTOR: Mammalian Target of Rapamycin
NSCLC: non-small cell lung cancer
EIF4EBP1: eukaryotic initiation factor 4E binding protein 1
p70S6K: p70 ribosomal protein S6-kinase
PARP: poly ADP-ribose polymerase.
